# Identification of a Novel Risk Locus for Multiple Sclerosis at 13q31.3 by a Pooled Genome-Wide Scan of 500,000 Single Nucleotide Polymorphisms

**DOI:** 10.1371/journal.pone.0003490

**Published:** 2008-10-22

**Authors:** Manuel Comabella, David W. Craig, Montse Camiña-Tato, Carlos Morcillo, Cristina Lopez, Arcadi Navarro, Jordi Rio, BiomarkerMS Study Group, Xavier Montalban, Roland Martin

**Affiliations:** 1 Unitat de Neuroimmunologia Clínica (UNIC), CEM-Cat, Hospital Universitari Vall d'Hebron (HUVH), Barcelona, Spain; 2 Neurogenomics Division, Translational Genomics Research Institute (TGen), Phoenix, Arizona, United States of America; 3 Departament de Ciencies Experimentals i de la Salut, Universitat Pompeu Fabra, Barcelona, Spain; 4 National Institute for Bioinformatics (INB), Barcelona, Spain; 5 Institució Catalana de Recerca i Estudis Avançats (ICREA), Barcelona, Spain; 6 National Institute of Neurological Disorders and Stroke, National Institutes of Health (NIH), Bethesda, Maryland, United States of America; Baylor College of Medicine, United States of America

## Abstract

Multiple sclerosis is a chronic inflammatory demyelinating disease of the central nervous system with an important genetic component and strongest association driven by the HLA genes. We performed a pooling-based genome-wide association study of 500,000 SNPs in order to find new loci associated with the disease. After applying several criteria, 320 SNPs were selected from the microarrays and individually genotyped in a first and independent Spanish Caucasian replication cohort. The 8 most significant SNPs validated in this cohort were also genotyped in a second US Caucasian replication cohort for confirmation. The most significant association was obtained for SNP rs3129934, which neighbors the HLA-DRB/DQA loci and validates our pooling-based strategy. The second strongest association signal was found for SNP rs1327328, which resides in an unannotated region of chromosome 13 but is in linkage disequilibrium with nearby functional elements that may play important roles in disease susceptibility. This region of chromosome 13 has not been previously identified in MS linkage genome screens and represents a novel risk locus for the disease.

## Introduction

Multiple sclerosis (MS) is a chronic inflammatory demyelinating disease of the central nervous system mediated by T-cell responses to myelin antigens [1]. Epidemiological studies have demonstrated that genetic factors contribute to the development of MS and probably represent the most important etiological component, although environmental factors likely contribute as well to disease manifestation and phenotypic expression [2]. During the last two decades, many investigative teams have devoted substantial efforts to identifying the individual genes that confer susceptibility to MS, and the following main conclusions have evolved from this work. 1/ MS is a complex genetic disease with multiple genes contributing with probably modest effects to disease susceptibility. 2/ Among the suspected susceptibility genes, the HLA-class II region on chromosomal area 6p21 contributes by far the most to genetic susceptibility and has been confirmed in many genetic studies in MS. In Caucasians, the ethnic group with the highest prevalence of MS, it is particularly the HLA-class II genes, HLA-DRB1*1501, -DRB5*0101, -DQA1*0102, -DQB1*0602, that are associated with MS [3], [4]. 3/ Genetic studies aiming to identify additional genes that confer susceptibility to MS have been rather disappointing, as many of the candidate genes identified in one study were not confirmed in others. It has not been until recently that two genes located outside the HLA region, the interleukin-2 receptor α (*IL2RA*) and the interleukin-7 receptor α (*IL7RA*), have been proposed as strong candidates for MS susceptibility in several studies [5]–[7]. Although the *IL2RA* and *IL7RA* genes exert much weaker effects than the HLA-class II genes, associations of these two genes with the disease have been confirmed in several independent MS cohorts.

Identification of these genes occurred through the use of a genome-wide association study of several hundred thousand single nucleotide polymorphisms (SNPs) in a broadly selected Caucasian population. Due to the need to multiple-test correct for the large number of SNPs assayed, the false negative rate for associations is quite high. An alternate approach is a two-stage design whereby an associated gene is found in a narrow population by a genome-wide screen and then evaluated in other populations. This approach is most effective when the associated allele is enriched in the initial population, such as due to a population bottleneck, but still present in the outbred population. However, the large cost associated with individually genotyping hundreds of individuals is often cost-prohibitive for the initial population. A more cost-effective alternative to individually genotyping thousands of samples on high-density SNP microarrays is the use of pooling-based GWA studies [8]. Effectively, a pooling-based GWA study accomplishes many of the objectives of individual genotyping and specifically allows for identification of many of the SNPs with the largest allelic frequency differences. In particular for MS, we can validate the GWA study-design and pooling-based approach since the HLA locus is clearly and consistently associated with MS [3], [4].

In our case, the pooling-based approach is particularly attractive since it allows us to test for associations across the Spanish population at relatively low cost as compared to individual genotyping, hypothesizing that additional genetic loci involved in conferring risk of MS could be identified through pooling-based GWA studies in this highly unique population.

## Materials and Methods

### Spanish Caucasian original cohort

A total of 242 unrelated patients with relapse-onset MS visited at the outpatient clinic of the Unitat de Neuroimmunologia Clínica (UNIC) were included in the study. All subjects were of Spanish origin and satisfied Poser's criteria for clinically definite MS [9]. The control population comprised of 242 unrelated individuals recruited at the hospital transfusion centre, which serves the geographic area from where the patients were enrolled. The study was approved by the Ethics Committee of Vall d'Hebron University Hospital and informed consent was obtained from all participants involved in the study. A summary of demographic and clinical characteristics of MS patients and controls is shown in Table 1.

**Table 1:** Demographic and clinical characteristics of the Spanish MS patients and healthy controls involved in the study.

Characteristics	MS	HC
*Original Spanish cohort* *		
n	242	242
Female/male (% women)	161/81 (66.5%)	158/84 (65.3%)
Age (years)^a^	43.5 (11.0)	43.9 (12.3)
RRMS/SPMS (% RRMS)	184/58 (76.0%)	-
*Spanish replication cohort* **		
n	100	100
Female/male (% women)	67/33 (67.0%)	65/35 (65.0%)
Age (years)^a^	41.8 (10.3)	42.9 (12.6)
RRMS/SPMS (% RRMS)	80/20 (80.0%)	-
*US replication cohort* **		
n	275	275
Female/male (% women)	223/52 (81.0%)	220/55 (80.0%)
Age (years)^a^	37.6 (8.9)	38.0 (9.5)
RRMS/SPMS (% RRMS)	275/0 (100.0%)	-

*Refers to patients used for DNA pooling. **Refers to patients used for individual validation. ^a^Data are expressed as mean (standard deviation, SD). RRMS: relapsing-remitting multiple sclerosis; SPMS: secondary progressive multiple sclerosis; HC: healthy controls.

### Sample pooling and high-density SNP genotyping of the Spanish Caucasian original cohort

Prior to quantitation all DNA samples were checked for quality using 2% agarose gel electrophoresis, and obviously degraded samples were excluded from the pooling analysis. Individual genomic DNA concentration of each subject was determined in triplicate with the Quant-iT PicoGreen dsDNA Assay Kit (Invitrogen, Carlsbad, CA) according to the manufacturer's instructions. These triplicate values were used to calculate a median concentration for each individual DNA. Individual DNA samples were then added to their respective pools in equivalent molar amounts. Once created, each pool was diluted to 50 ng/µl with sterile water in preparation for genotyping. Samples were genotyped (or allelotyped) on 9 replicate Affymetrix 500 K EA arrays, following the Affymetrix protocols (www.affymetrix.com).

### Analysis of pooled data from the SNP arrays

Probe intensity data was directly read from CEL files and Relative Allele Signal (RAS) values were calculated for each quartet. RAS values correspond to the ratio of the A probe to the sum of the A and B probes (where A is the major allele, and B is the minor allele), and provide a quantitative index correlating to allele frequencies in pooled DNA. These values yield independent measures of different hybridization events and are consequently treated as individual data points.

We used a silhouette statistic to rank all genotyped SNPs, since there are six paired-probes per SNP. This particular test-statistics has been heuristically shown to perform well at identifying SNPs with large allelic frequency differences, without merging data from fundamentally different probes even if for the same SNP. Consequentially, this test-statistic was utilized as it avoids introducing unnecessary variance by averaging probe intensity data from probes with different hybridization properties. This silhouette statistic intuitively represents the mean of the distance of a point to all other points in its class versus points in the other class. Silhouette scores range from 1, where significant separation between data points has been achieved and cluster assignment can be made with confidence, to −1, where differences in allelic frequencies are less reliable. The calculation for a silhouette score is shown in equation (1):
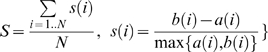
(1)


Here, the overall silhouette score, S, is the average of all of the individual silhouette values, s(i), for each of the measurements and N refers to the number of replicate measures. Shown in Equation 1, for a calculation of an s(i) value, b(i) refers to the mean distance of a point to all points not within its class and a(i) refers to the distance within its class. In this study, we have two classes, MS cases and matched controls. SNPs were ranked based on silhouette score, whereby the SNP with the highest score was ranked 1 and the SNP with the lowest score was ranked 428,867 using Affymetrix's Mendel3 libraries for the Affymetrix 500 K arrays. With this ranking, it is assumed that SNPs approaching a rank of 1 will have larger differences in allelic frequency.

### Validation of candidate SNPs from the arrays by individual genotyping in a first and independent Spanish Caucasian replication cohort

We applied two criteria to select candidate SNPs for validation in a replication cohort of cases and controls. A first criterion was to select the top 100 high-scoring SNPs from the arrays obtained after ranking SNPs using a silhouette statistic, as described above. A second criterion was to identify clusters of two or more SNPs located in close proximity and scoring in the top 1%, and subsequently select from each cluster the SNPs having the highest scores.

A Spanish Caucasian replication cohort comprised of one hundred unrelated relapse-onset MS patients attended at the outpatient clinic of the UNIC and one hundred controls from the hospital transfusion centre was used for validation of the selected top scoring SNPs. None of these MS patients and controls was part of the original cohort used for DNA pooling. Demographic and clinical characteristics of MS patients and controls are summarized in Table 1. Statistical significance of individual genotype data was calculated as an allelic χ^2^. This statistical model was chosen as it is analogous to the type of allelic associations that would detectable by a pooling-based GWA study.

DNA samples from this replication cohort were quantitated at the National Genotyping Center (CeGen, Madrid) using the PicoGreen assay. An Illumina GoldenGate assay was utilized to individually genotype SNPs selected from the Affymetrix 500 K platform.

Seven SNPs were discarded because of significant departures from Hardy-Weinberg equilibrium. Two samples were also discarded because their X chromosome genotypes were inconsistent with their reported sex.

Quality control processes and allelic and genotypic association tests were performed using the SNPator package (www.snpator.com) [10].

### US Caucasian replication cohort and SNP genotyping

A second replication cohort from the US and comprised of 275 unrelated relapse-onset MS patients and 275 controls was used to confirm association of the most significant SNPs validated in the Spanish replication cohort (those with p-values <0.01 following allelic frequency comparisons between cases and controls). A description of the demographic and clinical characteristics of the US replication cohort is shown in Table 1. DNA samples from this replication cohort were quantitated at TGen using the PicoGreen assay and individually genotyped for the selected SNPs by means of the TaqMan allelic discrimination assay.

## Results

A schematic flow chart summarizing the main steps of the study design and analysis is represented in Figure S1. A ranked list of 428,867 SNPs was obtained from analysis of the probe intensity differences between a pool of 242 Spanish relapse-onset MS individuals genotyped on 9 Affymetrix 500 K EA arrays and a pool of 242 Spanish healthy controls, also genotyped on 9 Affymetrix 500 K EA arrays. While a variety of analysis approaches can be conceived for detecting statistically significant differences in allelic frequency, we chose to implement an approach previously demonstrated effective at identifying the correct genetic loci in other pooling-based studies in complex diseases [8]. In this approach, separation of relative allele signals (RAS) between probe intensities for six unique probes between cases and controls are calculated using a silhouette test-statistic. This approach does not require that allele frequency be calculated from a large number of individually genotyped samples and does not assume that probes interrogate the same SNP with different 25 base oligos having the same hybridization properties. For the Affymetrix 500 K platform, this is a particular advantage since a SNP is probed by 6 to 10 probe pairs, each frequently with a different sequence (referred to as offset probes, where the SNP is not in the 13^th^ position). As expected, there has been evidence that some probes may poorly discriminate and some probes may preferentially bind one allele over another. The use of the silhouette statistics avoids adding unnecessary noise resulting from averaging fundamentally different measurements. The purpose of this paper is not to compare or contrast different methods, but rather to utilize a proven method to gain insight into the genetic basis of MS.

From the ranked list of SNPs and after applying the two selection criteria mentioned in Materials and Methods, a total of 320 SNPs were chosen for individual genotyping in a first and independent Spanish replication cohort of 100 MS cases and 100 healthy controls, in order to validate results from the pooled SNP arrays. We did not genotype in our original pooled cohorts since the purpose was not to evaluate pooling (as has been done elsewhere) and since the cost to do so would have increased the overall cost of the study to a level where individual genotyping would be more cost effective. Calculated association statistics are shown in Table 2 for the 8 SNPs showing greatest significance (p<0.01 after allelic frequency comparisons) and the full list is provided as Table S1. As expected in replicated association studies, most SNPs do not show association in the replication cohort.

**Table 2:** Association analysis of the 8 most significant SNPs that were validated in the Spanish replication cohort.

SNP	Location	Analysis	MS, N (%)	HC, N (%)	OR (95% CI)	P value
**rs3129934**	Chr. 6	Allele	n = 198	n = 186		
		C	133 (67.2%)	160 (86.0%)		
		**T**	65 (32.8%)	26 (14.0%)	3.0 (1.8–5.0)	1.4×10^−5^
		Genotype	n = 99	n = 93		
		CC	50 (50.5%)	69 (74.2%)		
		CT	33 (33.3%)	22 (23.7%)		
		TT	16 (16.2%)	2 (2.2%)	2.8 (1.5–5.2)	0.0007
**rs1327328**	Chr. 13	Allele	n = 198	n = 188		
		C	70 (35.4%)	96 (51.1%)		
		**T**	128 (64.6%)	92 (48.9%)	1.9 (1.3–2.9)	0.0018
		Genotype	n = 99	n = 94		
		CC	12 (12.1%)	29 (30.9%)		
		CT	46 (46.5%)	38 (40.4%)		
		TT	41 (41.0%)	27 (28.7%)	3.2 (1.5–6.8)	0.0015
**rs7141612**	Chr. 14	Allele	n = 198	n = 188		
		**C**	145 (73.2%)	112 (59.6%)		
		T	53 (26.8%)	76 (40.4%)	1.9 (1.2–2.9)	0.0045
		Genotype	n = 99	n = 94		
		CC	53 (53.5%)	35 (37.2%)		
		CT	39 (39.4%)	42 (44.7%)		
		TT	7 (7.1%)	17 (18.1%)	2.9 (1.1–7.4)	0.0205
**rs7821848**	Chr. 8	Allele	n = 198	n = 186		
		**C**	178 (89.9%)	148 (79.6%)		
		G	20 (10.1%)	38 (20.4%)	2.3 (1.3–4.1)	0.0047
		Genotype	n = 99	n = 93		
		CC	80 (80.8%)	58 (62.4%)		
		CG	18 (18.2%)	32 (34.4%)		
		GG	1 (1.0%)	3 (3.2%)	2.5 (1.3–4.9)	0.0045
**rs10925318**	Chr. 1	Allele	n = 198	n = 188		
		**C**	104 (52.5%)	72 (38.3%)		
		T	94 (47.5%)	116 (61.7%)	1.8 (1.2–2.7)	0.0050
		Genotype	n = 99	n = 94		
		CC	29 (29.3%)	17 (18.1%)		
		CT	46 (46.5%)	38 (40.4%)		
		TT	24 (24.2%)	39 (41.5%)	2.2 (1.2–4.1)	0.0106
**rs7326018**	Chr. 13	Allele	n = 198	n = 188		
		**A**	129 (65.2%)	96 (51.1%)		
		T	69 (34.8%)	92 (48.9%)	1.8 (1.2–2.9)	0.0050
		Genotype	n = 99	n = 94		
		AA	43 (43.4%)	28 (29.8%)		
		AT	43 (43.4%)	40 (42.6%)		
		TT	13 (13.1%)	26 (27.7%)	2.5 (1.2–5.3)	0.0120
**rs4902496**	Chr. 14	Allele	n = 198	n = 188		
		**C**	39 (19.7%)	18 (9.6%)		
		G	159 (80.3%)	170 (90.4%)	2.3 (1.3–4.2)	0.0051
		Genotype	n = 99	n = 94		
		CC	2 (2.0%)	2 (2.1%)		
		CG	35 (35.4%)	14 (14.9%)		
		GG	62 (62.6%)	78 (83.0%)	3.1 (1.6–6.3)	0.0011
**rs7204129**	Chr. 16	Allele	n = 198	n = 188		
		**C**	149 (75.3%)	117 (62.2%)		
		G	49 (24.7%)	71 (37.8%)	1.8 (1.2–2.9)	0.0057
		Genotype	n = 99	n = 94		
		CC	54 (54.5%)	34 (36.2%)		
		CG	41 (41.4%)	49 (52.1%)		
		GG	4 (4.0%)	11 (11.7%)	2.1 (1.2–3.8)	0.0104

Bold alleles denote risk alleles. Only the most statistically significant genotype comparisons are represented: rs3129934: CT+TT vs CC; rs1327328: CT+TT vs CC; rs7141612: CC+CT vs TT; rs7821848: CC vs CG+GG; rs10925318: CC+CT vs TT; rs732618: AA+AT vs TT; rs4902496: CG vs CC+GG; rs7204129: CC vs CG+GG.

**Figure 1:**
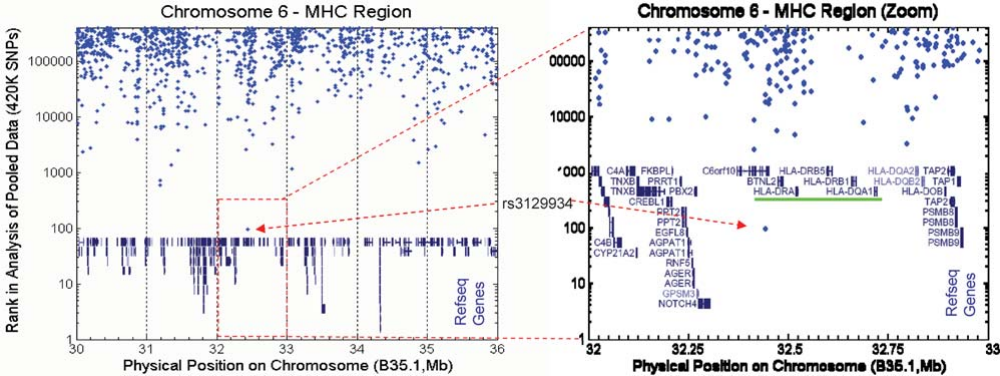
Graphs showing physical position of SNP rs3129934 on chromosome 6. SNP rs3129934, which showed the strongest association with the disease in the Spanish and US replication cohorts, is located approximately 200 kb from the HLA-DRB1 locus.

Our GWA study design and the pooling-based strategy is essentially validated by the fact that the single most significant SNP neighbors the HLA-DRB/DQA loci. Specifically, rs3129934 is approximately 200 kb from the HLA-DRB1 locus (Figure 1) and has a p-value of 1.4×10^−5^ with an OR of 3 (Table 2). Previous studies have identified linkage disequilibrium between HLA haplotypes and tagSNPs within the MHC [11]. Based on these data, rs3129934 is in very high linkage disequilibrium with SNP rs3135388 and demonstrated to tag the HLA-DRB associated haplotype in MS (D′ = 0.964 R^2^ = 0.93). The extremely strong linkage disequilibrium between these two markers allows us to conclude that the association of rs3129934 results from the HLA-DRB1 locus and that the association observed with rs3129934 does not represent a second MHC association signal.

The SNP rs1327328 has the next greatest association, with a p-value of 1.8×10^−3^ and an OR of 1.9 (Table 2). This SNP resides in a largely unannotated region of the genome. A second SNP (rs7326018) in this region also shows a p-value of 0.0050. Haplotype-based association analysis was completed using Haploview 3.32 for the two SNPs. In this analysis, four common haplotypes are observed, with significance being observed in the H1 and H2 haplotypes (Table 3).

**Table 3:** Haplotype analysis from SNPs rs7326018 and rs1237238 in the Spanish and US replication cohorts.

Haplotypes	Total, N	MS, N (%)	HC, N (%)	OR (95% CI)	P value
*Spanish replication cohort*					
(H1) AT	217	126 (63.6)	91 (48.4)	1.9 (1.2–2.8)	0.0026
(H2) TC	158	67 (33.8)	91 (48.4)	0.5 (0.4–0.8)	0.0036
(H3) AC	8	3 (1.5)	5 (2.7)	0.6 (0.1–2.4)	0.4302
(H4) TT	3	2 (1.0)	1 (0.5)	1.9 (0.2–21.2)	0.5928
*US replication cohort*					
(H1) AT	293	164 (61.9)	129 (54.4)	1.4 (1.0–1.7)	0.0293
(H2) TC	192	92 (34.7)	100 (42.2)	0.7 (0.4–1.0)	0.0116
(H3) AC	14	7 (2.6)	7 (3.0%)	0.9 (0.1–1.8)	0.0058
(H4) TT	3	2 (0.8)	1 (0.4%)	1.8 (0.1–3.6)	0.1701

First haplotype position refers to SNP rs7326018 and second position to SNP rs1237238. OR: odds ratio; 95% CI: 95% confidence interval.

Finally, five additional SNPs that were found to be associated with the disease in the Spanish replication cohort are the following (Table 2): rs7141612, intronic SNP in the *NPAS3* gene (neuronal PAS domain-containing protein 3); rs7821848, SNP located upstream of the *SNTG1* gene (syntrophin, gamma 1); rs10925318, intronic SNP in the *RYR2* gene (ryanodine receptor 2); rs4902496, SNP located in the 3'untranslated region of the *PLEKHH1* gene (pleckstrin homology domain containing, family H (with MyTH4 domain) member 1); rs7204129, intronic SNP in the *CDH13* gene (cadherin 13, H-cadherin (heart)).

Additional genotyping was conducted for those SNPs having highest significance in a new separate US Caucasian population consisting of up to 275 MS cases and 275 controls. The association statistics for these SNPs are shown in Table 4. The single most significant SNP, as expected, was rs3129934 neighboring the HLA-DRB1 locus with an allele-based p-value of 4.2×10^−10^. The second most significant SNP was rs1327328 on chromosome 13 with an allele based p-value of 0.0030, replicating the previously mentioned p-value of 1.8×10^−3^. For rs1327328, the association is driven by an excess of the T allele in both MS relapse-onset populations. The neighboring rs7326018 also showed significance at the 0.05 level, and as before showed some linkage disequilibrium with rs1327328 (D′ = 0.95, R^2^ = 0.91). Just as before, haplotype analysis revealed association of the H1 and H2 haplotypes with the disease (p = 0.03 and 0.01 respectively; see Table 3). The H3 haplotype also shows association at p = 0.006, though some caution must be used with interpretation due to the low frequency (2.1%) of this haplotype compared to the H1, H2, and H4 haplotypes. Beyond these three SNPs, no other of the 8 genotyped SNPs showed association in the new US replication cohort (Table 4).

**Table 4:** Association analysis of selected SNPs genotyped in the US Caucasian replication cohort.

SNP	Location	Analysis	MS, N (%)	HC, N (%)	OR (95% CI)	P value
**rs3129934**	Chr. 6	Allele	n = 550	n = 480		
		C	376 (68.4%)	408 (85.0%)		
		**T**	174 (31.6%)	72 (15.0%)	2.6 (1.9–3.6)	4.2×10^−10^
		Genotype	n = 275	n = 240		
		CC	124 (45.1%)	176 (73.3%)		
		CT	128 (46.5%)	56 (23.3%)		
		TT	23 (8.4%)	8 (3.3%)	3.3 (2.3–4.9)	9.0×10^−11^
**rs1327328**	Chr. 13	Allele	n = 548	n = 482		
		C	197 (35.9%)	217 (45.0%)		
		**T**	351 (64.1%)	265 (55.0%)	1.4 (1.1–1.9)	0.0030
		Genotype	n = 274	n = 241		
		CC	37 (13.5%)	47 (19.5%)		
		CT	123 (44.9%)	123 (51.0%)		
		TT	114 (41.6%)	71 (29.5%)	1.7 (1.2–2.5)	0.0042
**rs7141612**	Chr. 14	Allele	n = 544	n = 478		
		**C**	363 (66.7%)	300 (62.8%)		
		T	181 (33.3%)	178 (37.2%)	1.2 (0.9–1.5)	0.1851
		Genotype	n = 272	n = 239		
		CC	128 (47.1%)	99 (41.4%)		
		CT	107 (39.3%)	102 (42.7%)		
		TT	37 (13.9%)	38 (15.9%)	1.3 (0.9–1.8)	0.2008
**rs7821848**	Chr. 8	Allele	n = 552	n = 484		
		**C**	451 (81.7%)	389 (80.4%)		
		G	101 (18.3%)	95 (19.6%)	1.1 (0.8–1.5)	0.5852
		Genotype	n = 276	n = 242		
		CC	186 (67.4%)	159 (65.7%)		
		CG	79 (28.6%)	71 (29.3%)		
		GG	11 (4.0%)	12 (5.0%)	1.3 (0.5–2.9)	0.5916
**rs10925318**	Chr. 1	Allele	n = 556	n = 486		
		**C**	296 (53.2%)	247 (50.8%)		
		T	260 (46.8%)	239 (49.2%)	1.1 (0.9–1.4)	0.4364
		Genotype	n = 278	n = 243		
		CC	77 (27.7%)	59 (24.3%)		
		CT	142 (51.1%)	129 (53.1%)		
		TT	59 (21.2%)	55 (22.6%)	1.2 (0.8–1.8)	0.3755
**rs7326018**	Chr. 13	Allele	n = 536	n = 478		
		**A**	338 (63.1%)	273 (57.1%)		
		T	198 (36.9%)	205 (42.9%)	1.3 (1.0–1.6)	0.0534
		Genotype	n = 268	n = 239		
		AA	102 (38.1%)	80 (33.5%)		
		AT	134 (50.0%)	113 (47.3%)		
		TT	32 (11.9%)	46 (19.2%)	1.8 (1.1–2.9)	0.0228
**rs4902496**	Chr. 14	Allele	n = 540	n = 486		
		C	99 (18.3%)	105 (21.6%)		
		**G**	441 (81.7%)	381 (78.4%)	1.2 (0.9–1.7)	0.1899
		Genotype	n = 270	n = 243		
		CC	11 (4.1%)	10 (4.1%)		
		CG	77 (28.5%)	85 (35.0%)		
		GG	182 (67.4%)	148 (60.9%)	1.3 (0.9–2.0)	0.1160
**rs7204129**	Chr. 16	Allele	n = 198	n = 188		
		**C**	356 (65.4%)	306 (63.5%)		
		G	188 (34.6%)	176 (36.5%)	1.1 (0.8–1.4)	0.5135
		Genotype	n = 272	n = 241		
		CC	109 (40.1%)	97 (40.2%)		
		CG	138 (50.7%)	112 (46.5%)		
		GG	25 (9.2%)	32 (13.3%)	1.5 (0.9–2.6)	0.1416

Bold alleles denote risk alleles. Genotype comparisons: rs3129934: TT+CT vs CC; rs1327328: TT vs CT+CC; rs7141612: CC vs CT+TT; rs7821848: CC+CG vs GG; rs10925318: CC vs CT+TT; rs732618: AA+AT vs TT; rs4902496: CC+GG vs CG; rs7204129: CC+CG vs GG.

SNP rs1327328 was genotyped in an additional US Caucasian population of 178 MS cases (mean age (standard deviation): 37.0 (9.9); % women: 81%; % relapsing-remitting MS: 100%) and 658 controls (mean age: 38.0 (11.9); % women: 80% female). Table 5 depicts allele and genotype associations obtained for the combined Caucasian replications cohorts comprised of up to 553 MS cases and 1033 controls. Both allele T (OR = 1.3; 95% CI = 1.1 to 1.5; p = 5.0×10^−4^) and TT homozygosity (OR = 1.4; 95% CI = 1.1 to 1.8; p = 1.7×10^−3^) for SNP rs1327328 were associated with relapse-onset MS. Individual association analysis obtained for the whole US replication cohort is also shown in Table 5.

**Table 5:** Association analysis of SNP rs1327328 in the combined Spanish and US replication cohorts.

	Analysis	MS, N (%)	HC, N(%)	OR (95% CI)	P value
Combined*	Allele	n = 1106	n = 2066		
	C	426 (38.5)	927 (44.9)		
	**T**	680 (61.5)	1139 (55.1)	1.3 (1.1–1.5)	5.0×10^−4^
	Genotype	n = 553	n = 1033		
	CC	85 (15.4)	208 (20.1)		
	CT	256 (46.3)	511 (49.5)		
	TT	212 (38.3)	314 (30.4)	1.4 (1.1–1.8)	1.7×10^−3^
US cohort**	Allele	n = 908	n = 1878		
	C	356 (39.2)	831 (44.2)		
	**T**	552 (60.8)	1047 (55.8)	1.2 (1.0–1.4)	0.0116
	Genotype	n = 454	n = 939		
	CC	73 (16.1)	179 (19.1)		
	CT	210 (46.3)	473 (50.4)		
	TT	171 (37.7)	287 (30.6)	1.4 (1.1–1.7)	0.0082

*Meta-analysis for pooled cohorts (Spanish and US) was performed using the Mantel-Haenszel test implemented in SAS 9.0. **Refers to the individual results obtained for the whole US replication cohort. Risk alleles are shown in bold. Only the most statistically significant genotype comparisons are represented (TT vs CC+CT in both analyses). OR: odds ratio; 95% CI: 95% confidence interval.

**Figure 2:**
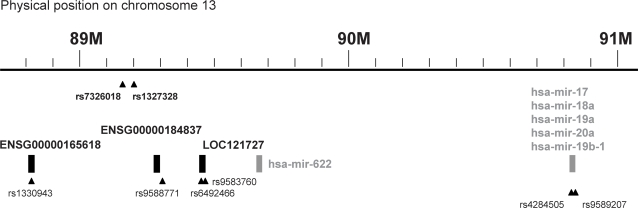
Graph illustrating genes located in proximity to SNPs rs1327328 and rs7326018 on chromosome 13. SNP rs1327328 was the second most significant SNP validated in the Spanish and US replication cohorts, and is in strong linkage disequilibrium with SNP rs7326018. SNPs rs1330943, rs9588771, rs6492466, rs9583760, rs4284505, and rs9589207 have been typed by HapMap.

As mentioned above, SNPs rs1327328 and rs7326018 lie in a position of chromosome 13 devoid of any known functional elements. As shown in Figure 2, the region were these SNPs are located contains several genes (*ENSG00000184837*, *ENSG00000165618* and *LOC121727*) and microRNAs (*hsa-mir-622*, *hsa-mir-17*, *hsa-mir-18a*, *hsa-mir-19a*, *hsa-mir-20a* and *hsa-mir-19b-1*). Within or close to some of these functional elements, there are SNPs that have been typed by HapMap. Linkage disequilibrium analysis between SNPs rs1327328 and rs7326018 and the SNPs mapping in the functional elements is summarized in Table 6. Linkage disequilibrium measures are highly significant between SNPs rs1327328 and rs7326018 and SNP rs4284505, located next to a cluster of microRNAs, and marginally significant with SNP rs9583760, located within *LOC121727* (similar to Peroxisome assembly protein 12 (Peroxin-12) (Peroxisome assembly factor 3) (PAF-3)).

**Table 6:** Linkage disequilibrium analysis between SNPs rs7326018 and rs1327328 and neighboring SNPs in chromosome 13.

SNP pairs		D′	r^2^	Chi^2^	P value
rs7326018	rs1330943	0.3836	0.0023	0.4106	0.5217
	rs1327328	0.9539	0.9099	163.7904	1.89×10^−15^
	rs9588771	0.0000	-	0	1
	rs6492466	0.0563	0.0014	0.2467	0.6194
	rs9583760	0.2725	0.0282	5.0707	**0.0243**
	rs4284505	0.5002	0.1192	21.4486	**3.63×10^−6^**
	rs9589207	1.0000	0.0165	2.9644	0.0851
rs1327328	rs1330943	0.3836	0.0023	0.4106	0.5217
	rs9588771	0.0000	-	0	1
	rs6492466	0.0563	0.0014	0.2467	0.6194
	rs9583760	0.2725	0.0282	5.0707	**0.0243**
	rs4284505	0.5668	0.153	27.5447	**1.54×10^−7^**
	rs9589207	1.0000	0.0165	2.9644	0.0851

SNPs rs1330943 and rs9588771 are located in *ENSG00000165618* and *ENSG00000184837* genes respectively; rs6492466 and rs9583760 are located within *LOC121727*; rs4284505 is located close to a cluster of microRNAs and rs9589207 within the *hsa-mir-92a-1* microRNA. Linkage disequilibrium measures are highly significant between SNPs rs1327328 and rs7326018 and SNP rs4284505, and marginally significant with SNP rs9583760 (p values are represented in bold).

## Discussion

Despite a large number of studies on susceptibility genes for MS, these efforts during the last two decades have been notably disappointing. Several whole genome screens and many linkage and association studies have only shown one genetic region with high consistency, the HLA-class II region on 6p21 and in Caucasian MS patients [3], [4]. Recently, two cytokine receptors, *IL7RA* and *IL2RA*, have been shown to contribute to the non-HLA-related susceptibility to MS [5]–[7]. Nevertheless, MS is a complex trait disease and additional loci are expected to be contributing to the genetic risk in MS.

In the present study we used a recently developed and validated DNA pooling strategy and SNP array genotyping of 9 replicate pools as a first step. As the main advantage, this approach allows cutting costs and hence permitted us to perform the study on a novel population in order to screen a large number of SNPs for allelic association. Due to the large number of SNPs some correction is necessary in order to account for multiple testing. We did not genotype our original population, and all p-values are obtained from new cohorts that are completely non-redundant with the original pooled populations. Under the most conservative of scenarios, the p-values reported in Table 2 must survive a Bonferroni-corrected significance level of p = 1.5×10^−4^. This is an overly conservative number since some SNPs do show linkage disequilibrium between each other and, thus, the assumption of test independence central to the Bonferroni correction is not met. Still, rs3129934 survives this Bonferroni corrected significance level and, likewise, is known to be in strong linkage disequilibrium with markers in the HLA-DRB1 locus [11]. As fully expected, rs3129934 showed also the highest association in the US Caucasian replication cohort. These findings validate the pooling-based strategy that has been used in our study. Several studies have shown that MS is primarily associated with the DR2 haplotype (DRB1*1501-DQB1*0602) in the Spanish population. In addition, our results are in agreement with previous publications suggesting that the effect of HLA is less significant in the Spanish population compared with other US populations with higher disease incidence [12], [13].

SNP rs1327328 showed the second strongest association with MS in the Spanish replication cohort with a p-value of 1.8×10^−3^. A second SNP, rs7326018, located approximately 30 kb from rs1327328 and in strong linkage disequilibrium with the latter was also associated with the disease in this replication cohort. The fact that both rs1327328 and rs7326018 show strong evidence for association suggests that genotyping error is not leading to a false association. SNP rs1327328 was validated in the US Caucasian population, with an allele-based p-value of 0.003, below the 0.006 Bonferroni-corrected significance threshold.

SNPs rs1327328 and rs7326018 are located in the long arm of chromosome 13, at 13q31.3, in a region that has not been previously identified in MS linkage genome screens. In addition, rs1327328 and rs7326018 have not been found associated with the disease in other GWA studies. Thus, this region represents a novel risk locus for the disease. As mentioned before, this region of chromosome 13 is largely unannotated with no functional elements in close proximity. As to what could be the cause of the associations detected for these SNPs, a possibility is that they are acting as markers of some nearby elements. Interestingly, rs1327328 and rs7326018 are in high linkage disequilibrium with other SNPs mapping within or close to a cluster of microRNAs. MicroRNAs are a class of small regulatory RNAs that regulate gene expression [14], and polymorphisms located within or in proximity to microRNAs may contribute to disease susceptibility [15]. Several members of this microRNA cluster found to be in linkage disequilibrium with the MS-associated SNPs rs1327328 and rs7326018 are known to negatively regulate the expression of the transcription factor *E2F1* [16]. The E2F transcription factor family, with *E2F1* as the best characterized member, plays a crucial role in the control of cell cycle [17]. Of note, the E2F pathway has been implicated in autoimmunity [18], and a gene expression profiling study with microarrays found an overrepresentation of the E2F pathway in PBMC from relapse-onset MS patients when compared with healthy controls [19].

Linkage disequilibrium is also significant between SNPs rs1327328 and rs7326018 and markers mapping within or close to locus *LOC121727*. This locus is a possible gene with homology to the peroxisomal biogenesis factor 12 (*PEX12*). Of note, mutations in human *PEX12* result in Zellweger syndrome [20], [21], one of a group of several related diseases called peroxisome biogenesis disorders, which are part of a larger group of diseases known as the leukodystrophies [22]. These findings suggest that nearby elements in linkage disequilibrium with SNPs rs1327328 and rs7326018 may be playing important roles in MS susceptibility.

Finally, 5 of the 8 SNPs that were validated in the Spanish replication cohort were not associated with the disease in the US population. Regarding the ethnic background of the MS patients that were part of the replication cohorts, they are all Caucasian, however, one probably has to consider different ethnic admixtures in the US Caucasian and the Spanish Caucasian MS cohorts. Additionally, there are regional differences between MS prevalence and it is possible that susceptibility alleles for MS in a Spanish MS population may not be prevalent in a US Caucasian population. Consequentially, failure to see association of the other SNPs does not provide definitive evidence of a negative association, and these SNPs may represent regions of potential susceptibility to MS in the Spanish population. Furthermore, none of the 8 SNPs validated in the Spanish replication cohort by individual genotyping were found associated with the disease in a recent genomewide study conducted by the International Multiple Sclerosis Genetics Consortium [7].

In summary, by using a pooling-based approach and high density SNP arrays we confirmed the well-known association of the HLA class II genes with the disease, and identified a novel risk locus for MS on chromosome 13. Nevertheless, these findings are of preliminary nature and future studies will be needed to validate these results on chromosome 13 and to further implicate the functional elements of this region in disease susceptibility.

## Supporting Information

Figure S1 Flow chart summarizing the different steps undertaken in the analysis of the study data. A ranked list of SNPs was obtained from the analysis of the SNP arrays performed on pooled DNA from 242 Spanish MS cases and 242 Spanish controls (original Spanish cohort). After applying two criteria (arrows), 320 SNPs were selected for individual genotyping in an independent Spanish cohort of 100 MS cases and 100 controls (Spanish replication cohort (1)). The SNPs showing greatest significance (p<0.01 after allelic frequency comparisons) were genotyped in a US cohort comprised of 275 MS cases and 275 controls (US replication cohort (2)). Two out of 8 SNPs (rs3129934 and rs1327328) were validated in the US cohort. A third SNP (rs7326018) showed marginal association in the US cohort. SNP rs3129934 is located in close proximity to the HLA-DRB1 locus. SNP rs1327328 was genoptyped in an additional US cohort of 178 MS cases and 658 controls and is located in chromosome 13, in a region that represents a novel risk locus for MS.
(0.69 MB TIF)

Table S1 Association analysis of the remaining 312 SNPs selected from the pooled SNP arrays and genotyped in the Spanish replication cohort.
(0.46 MB DOC)
