# Supplementary figures and images for: Identification of a Novel Risk Locus for Multiple Sclerosis at 13q31.3 by a Pooled Genome-Wide Scan of 500,000 Single Nucleotide Polymorphisms
Source: PLoS One. 2008 Oct 22;3(10):e3490. doi: 10.1371/journal.pone.0003490 (PMC2566815; doi:10.1371/journal.pone.0003490)

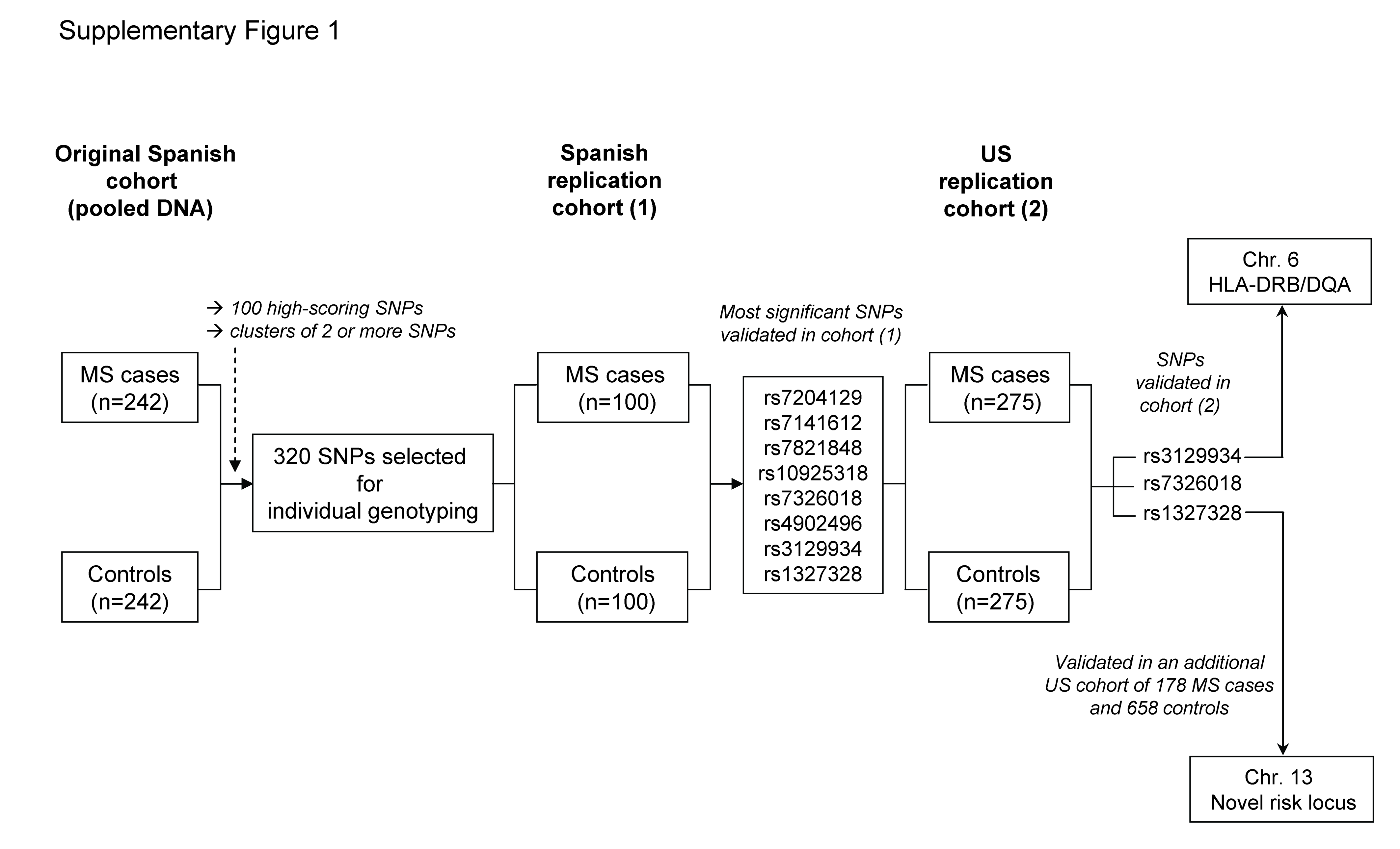

Supplement: Figure S1 — Flow chart summarizing the different steps undertaken in the analysis of the study data. A ranked list of SNPs was obtained from the analysis of the SNP arrays performed on pooled DNA from 242 Spanish MS cases and 242 Spanish controls (original Spanish cohort). After applying two criteria (arrows), 320 SNPs were selected for individual genotyping in an independent Spanish cohort of 100 MS cases and 100 controls (Spanish replication cohort (1)). The SNPs showing greatest significance (p<0.01 after allelic frequency comparisons) were genotyped in a US cohort comprised of 275 MS cases and 275 controls (US replication cohort (2)). Two out of 8 SNPs (rs3129934 and rs1327328) were validated in the US cohort. A third SNP (rs7326018) showed marginal association in the US cohort. SNP rs3129934 is located in close proximity to the HLA-DRB1 locus. SNP rs1327328 was genoptyped in an additional US cohort of 178 MS cases and 658 controls and is located in chromosome 13, in a region that represents a novel risk locus for MS. (0.69 MB TIF) [file pone.0003490.s001.tif]
