# Supplementary material for: Identification of a Novel Risk Locus for Multiple Sclerosis at 13q31.3 by a Pooled Genome-Wide Scan of 500,000 Single Nucleotide Polymorphisms
Source: PLoS One. 2008 Oct 22;3(10):e3490. doi: 10.1371/journal.pone.0003490 (PMC2566815; doi:10.1371/journal.pone.0003490)
Supplement: Table S1 — Association analysis of the remaining 312 SNPs selected from the pooled SNP arrays and genotyped in the Spanish replication cohort. (0.46 MB DOC) [file pone.0003490.s002.doc]

**Table S1.** Association analysis of the remaining 312 SNPs selected from the pooled SNP arrays and genotyped in the Spanish replication cohort.

| SNP | Location | Risk allele | OR | 95% IC | P value |
| --- | --- | --- | --- | --- | --- |
| rs11659205 | Chr. 18 | G | 1.91 | 1.14 - 3.23 | 0.0138 |
| rs10834707 | Chr. 11 | C | 1.80 | 1.10 - 2.97 | 0.0195 |
| rs7703938 | Chr. 5 | A | 2.14 | 1.10 - 4.14 | 0.0224 |
| rs17012337 | Chr. 2 | G | 1.81 | 1.08 - 3.04 | 0.0232 |
| rs9436678 | Chr. 1 | T | 1.66 | 1.05 - 2.62 | 0.0281 |
| rs11981782 | Chr. 7 | C | 1.57 | 1.04 - 2.37 | 0.0300 |
| rs235770 | Chr. 20 | C | 1.58 | 1.04 - 2.40 | 0.0306 |
| rs2236241 | Chr. 14 | C | 1.75 | 1.04 - 2.97 | 0.0349 |
| rs2749484 | Chr. 14 | C | 1.67 | 1.03 - 2.68 | 0.0353 |
| rs1390840 | Chr. 18 | G | 1.56 | 1.03 - 2.35 | 0.0356 |
| rs5987077 | Chr. X | A | 1.81 | 1.03 - 3.16 | 0.0364 |
| rs6584632 | Chr. 10 | G | 1.54 | 1.03 - 2.31 | 0.0372 |
| rs6865460 | Chr. 5 | T | 9.68 | 0.52 - 181.10 | 0.0391 |
| rs4835680 | Chr. 5 | A | 1.53 | 1.01 - 2.31 | 0.0442 |
| rs790660 | Chr. 10 | T | 1.51 | 1.01 - 2.26 | 0.0446 |
| rs235771 | Chr. 20 | T | 1.52 | 1.00 - 2.30 | 0.0474 |
| rs28488 | Chr. 20 | T | 1.53 | 1.00 - 2.32 | 0.0474 |
| rs12198616 | Chr. 6 | C | 1.52 | 1.00 - 2.29 | 0.0486 |
| rs6823779 | Chr. 4 | A | 1.51 | 1.00 - 2.28 | 0.0509 |
| rs3127188 | Chr. 6 | A | 1.49 | 1.00 - 2.23 | 0.0522 |
| rs17279083 | Chr. 7 | G | 1.51 | 0.99 - 2.30 | 0.0566 |
| rs7152838 | Chr. 14 | G | 1.52 | 0.98 - 2.35 | 0.0594 |
| rs10213884 | Chr. 5 | C | 1.48 | 0.98 - 2.22 | 0.0607 |
| rs2345444 | Chr. 16 | C | 1.76 | 0.96 - 3.21 | 0.0639 |
| rs1942200 | Chr. 18 | G | 2.02 | 0.94 - 4.35 | 0.0663 |
| rs1726427 | Chr. 12 | C | 1.49 | 0.97 - 2.30 | 0.0672 |
| rs969930 | Chr. 8 | G | 1.46 | 0.96 - 2.20 | 0.0730 |
| rs1941575 | Chr. 18 | G | 1.50 | 0.95 - 2.37 | 0.0790 |
| rs850548 | Chr. 7 | G | 1.47 | 0.95 - 2.26 | 0.0828 |
| rs4278097 | Chr. 7 | A | 1.48 | 0.93 - 2.35 | 0.0989 |
| rs7996840 | Chr. 13 | C | 1.49 | 0.92 - 2.42 | 0.1048 |
| rs6559754 | Chr. 9 | G | 1.40 | 0.93 - 2.09 | 0.1064 |
| rs1941566 | Chr. 18 | G | 1.52 | 0.90 - 2.56 | 0.1127 |
| rs10814997 | Chr. 9 | C | 1.42 | 0.92 - 2.20 | 0.1132 |
| rs13080133 | Chr. 3 | C | 1.38 | 0.92 - 2.06 | 0.1160 |
| rs4805721 | Chr. 19 | T | 1.38 | 0.92 - 2.07 | 0.1166 |
| rs2669845 | Chr. 3 | T | 1.53 | 0.89 - 2.63 | 0.1236 |
| rs17109366 | Chr. 14 | G | 1.48 | 0.89 - 2.44 | 0.1288 |
| rs12918211 | Chr. 16 | A | 1.36 | 0.91 - 2.03 | 0.1345 |
| rs6578027 | Chr. 8 | C | 1.39 | 0.90 - 2.15 | 0.1362 |
| rs8086036 | Chr. 18 | T | 1.36 | 0.91 - 2.03 | 0.1366 |
| rs10845572 | Chr. 12 | C | 1.36 | 0.91 - 2.03 | 0.1377 |
| rs821592 | Chr. 1 | C | 1.43 | 0.89 - 2.28 | 0.1389 |
| rs13197267 | Chr. 6 | G | 1.35 | 0.90 - 2.03 | 0.1414 |
| rs3105791 | Chr. 5 | G | 1.37 | 0.90 - 2.10 | 0.1434 |
| rs3846161 | Chr. 3 | T | 1.40 | 0.89 - 2.21 | 0.1443 |
| rs2460689 | Chr. 6 | G | 1.34 | 0.90 - 2.01 | 0.1476 |
| rs546439 | Chr. 11 | C | 1.38 | 0.89 - 2.14 | 0.1483 |
| SNP | Location | Risk allele | OR | 95% IC | P value |
| rs1966252 | Chr. 7 | G | 1.37 | 0.89 - 2.10 | 0.1498 |
| rs606911 | Chr. 17 | A | 1.53 | 0.85 - 2.77 | 0.1544 |
| rs11052430 | Chr. 12 | G | 1.82 | 0.78 - 4.27 | 0.1626 |
| rs11666638 | Chr. 19 | A | 1.34 | 0.89 - 2.01 | 0.1666 |
| rs9569549 | Chr. 13 | T | 1.36 | 0.88 - 2.11 | 0.1675 |
| rs1888567 | Chr. 14 | A | 1.35 | 0.88 - 2.06 | 0.1701 |
| rs7959282 | Chr. 12 | T | 1.34 | 0.88 - 2.04 | 0.1708 |
| rs9568313 | Chr. 13 | A | 1.32 | 0.88 - 1.97 | 0.1745 |
| rs2591732 | Chr. 5 | C | 1.33 | 0.88 - 2.00 | 0.1786 |
| rs1374019 | Chr. 5 | T | 1.33 | 0.88 - 2.00 | 0.1786 |
| rs12133753 | Chr. 1 | C | 1.46 | 0.84 - 2.54 | 0.1794 |
| rs11130855 | Chr. 3 | T | 1.36 | 0.87 - 2.13 | 0.1799 |
| rs6979640 | Chr. 7 | C | 1.32 | 0.88 - 2.00 | 0.1804 |
| rs301568 | Chr. 3 | G | 1.39 | 0.85 - 2.27 | 0.1834 |
| rs2284372 | Chr. 20 | A | 1.43 | 0.84 - 2.43 | 0.1891 |
| rs2497666 | Chr. 10 | A | 1.53 | 0.81 - 2.91 | 0.1892 |
| rs9276440 | Chr. 6 | A | 1.31 | 0.87 - 1.98 | 0.1959 |
| rs3774854 | Chr. 4 | G | 1.30 | 0.86 - 1.95 | 0.2135 |
| rs12580432 | Chr. 12 | C | 1.35 | 0.84 - 2.15 | 0.2147 |
| rs1880055 | Chr. 10 | C | 1.30 | 0.86 - 1.96 | 0.2168 |
| rs12220353 | Chr. 10 | C | 1.29 | 0.86 - 1.92 | 0.2189 |
| rs10890507 | Chr. 1 | T | 1.30 | 0.85 - 2.00 | 0.2213 |
| rs2615997 | Chr. 1 | T | 2.26 | 0.58 - 8.87 | 0.2305 |
| rs7222683 | Chr. 17 | G | 1.30 | 0.84 - 1.99 | 0.2347 |
| rs4862699 | Chr. 4 | G | 1.30 | 0.84 - 2.00 | 0.2352 |
| rs6840697 | Chr. 4 | T | 1.30 | 0.84 - 2.00 | 0.2352 |
| rs3753955 | Chr. 1 | C | 1.36 | 0.82 - 2.28 | 0.2363 |
| rs1886933 | Chr. 13 | G | 1.27 | 0.85 - 1.91 | 0.2370 |
| rs3807154 | Chr. 7 | C | 1.30 | 0.84 - 2.02 | 0.2459 |
| rs855325 | Chr. 1 | G | 1.29 | 0.84 - 1.99 | 0.2476 |
| rs1422902 | Chr. 5 | G | 1.28 | 0.84 - 1.96 | 0.2480 |
| rs12819116 | Chr. 12 | A | 1.28 | 0.84 - 1.96 | 0.2520 |
| rs2038963 | Chr. 9 | T | 1.33 | 0.81 - 2.16 | 0.2568 |
| rs17065434 | Chr. 5 | C | 1.31 | 0.82 - 2.11 | 0.2606 |
| rs2242381 | Chr. 12 | G | 1.31 | 0.82 - 2.10 | 0.2617 |
| rs2028472 | Chr. 5 | C | 1.30 | 0.82 - 2.07 | 0.2625 |
| rs9915613 | Chr. 17 | A | 1.27 | 0.83 - 1.94 | 0.2643 |
| rs390436 | Chr. 10 | G | 1.27 | 0.83 - 1.95 | 0.2648 |
| rs17415114 | Chr. 2 | A | 1.29 | 0.82 - 2.03 | 0.2656 |
| rs3851075 | Chr. 10 | G | 1.25 | 0.83 - 1.87 | 0.2800 |
| rs7738608 | Chr. 6 | C | 1.30 | 0.80 - 2.10 | 0.2874 |
| rs2282604 | Chr. 11 | A | 1.24 | 0.83 - 1.87 | 0.2975 |
| rs9856066 | Chr. 3 | G | 1.23 | 0.83 - 1.84 | 0.3060 |
| rs10952742 | Chr. 7 | T | 1.24 | 0.82 - 1.89 | 0.3073 |
| rs4377167 | Chr. 16 | T | 1.26 | 0.81 - 1.96 | 0.3074 |
| rs1789547 | Chr. 18 | G | 1.24 | 0.82 - 1.89 | 0.3111 |
| rs8138968 | Chr. 22 | C | 1.24 | 0.81 - 1.90 | 0.3129 |
| rs2274061 | Chr. 13 | G | 1.28 | 0.79 - 2.07 | 0.3141 |
| rs12126366 | Chr. 1 | C | 1.88 | 0.54 - 6.51 | 0.3148 |
| rs3793260 | Chr. 7 | C | 1.74 | 0.57 - 5.30 | 0.3219 |
| rs2345955 | Chr. 2 | T | 1.30 | 0.77 - 2.18 | 0.3241 |
| rs996609 | Chr. 7 | C | 1.23 | 0.81 - 1.85 | 0.3298 |
| rs6938971 | Chr. 6 | G | 1.29 | 0.76 - 2.18 | 0.3378 |
| SNP | Location | Risk allele | OR | 95% IC | P value |
| rs2494731 | Chr. 14 | G | 1.22 | 0.81 - 1.85 | 0.3427 |
| rs2480455 | Chr. 9 | A | 1.22 | 0.80 - 1.86 | 0.3465 |
| rs10427669 | Chr. 22 | A | 1.22 | 0.80 - 1.86 | 0.3477 |
| rs9313730 | Chr. 5 | A | 1.21 | 0.81 - 1.81 | 0.3495 |
| rs7543435 | Chr. 1 | A | 1.21 | 0.81 - 1.83 | 0.3507 |
| rs17286411 | Chr. 16 | G | 1.21 | 0.81 - 1.83 | 0.3507 |
| rs4761588 | Chr. 12 | A | 1.21 | 0.81 - 1.83 | 0.3541 |
| rs1535228 | Chr. 6 | T | 1.34 | 0.72 - 2.52 | 0.3546 |
| rs17002173 | Chr. 21 | A | 1.30 | 0.74 - 2.27 | 0.3556 |
| rs460781 | Chr. 5 | T | 1.46 | 0.65 - 3.26 | 0.3562 |
| rs16863396 | Chr. 3 | A | 1.21 | 0.80 - 1.83 | 0.3573 |
| rs4787291 | Chr. 16 | G | 1.29 | 0.75 - 2.24 | 0.3584 |
| rs3125704 | Chr. 13 | C | 1.21 | 0.80 - 1.84 | 0.3616 |
| rs6956225 | Chr. 7 | C | 1.28 | 0.75 - 2.19 | 0.3631 |
| rs892277 | Chr. 3 | C | 1.22 | 0.79 - 1.86 | 0.3700 |
| rs6678270 | Chr. 1 | C | 1.26 | 0.76 - 2.08 | 0.3721 |
| rs2246209 | Chr. 1 | A | 1.23 | 0.78 - 1.93 | 0.3776 |
| rs2332059 | Chr. 1 | T | 1.20 | 0.80 - 1.81 | 0.3820 |
| rs2548724 | Chr. 5 | A | 1.25 | 0.76 - 2.07 | 0.3830 |
| rs9387522 | Chr. 6 | C | 1.20 | 0.79 - 1.82 | 0.3840 |
| rs11158927 | Chr. 14 | C | 1.27 | 0.74 - 2.18 | 0.3853 |
| rs12773063 | Chr. 10 | G | 1.19 | 0.80 - 1.78 | 0.3932 |
| rs11633256 | Chr. 15 | A | 1.33 | 0.69 - 2.56 | 0.4001 |
| rs2829276 | Chr. 21 | C | 1.19 | 0.79 - 1.80 | 0.4016 |
| rs9321643 | Chr. 6 | T | 1.22 | 0.77 - 1.95 | 0.4017 |
| rs7920704 | Chr. 10 | A | 1.20 | 0.78 - 1.86 | 0.4041 |
| rs7076566 | Chr. 10 | A | 1.22 | 0.76 - 1.94 | 0.4063 |
| rs2460627 | Chr. 15 | C | 1.30 | 0.69 - 2.45 | 0.4102 |
| rs17422753 | Chr. 6 | C | 1.25 | 0.73 - 2.15 | 0.4111 |
| rs1075174 | Chr. 20 | T | 1.19 | 0.78 - 1.82 | 0.4134 |
| rs2259816 | Chr. 12 | C | 1.19 | 0.79 - 1.79 | 0.4139 |
| rs1399626 | Chr.2 | T | 1.19 | 0.78 - 1.80 | 0.4175 |
| rs632632 | Chr. 2 | C | 1.18 | 0.79 - 1.76 | 0.4183 |
| rs10788882 | Chr. 1 | C | 1.19 | 0.78 - 1.80 | 0.4210 |
| rs17606174 | Chr. 6 | T | 1.30 | 0.68 - 2.49 | 0.4219 |
| rs9422941 | Chr. 10 | A | 1.26 | 0.71 - 2.22 | 0.4307 |
| rs16904185 | Chr. 8 | G | 1.19 | 0.77 - 1.85 | 0.4316 |
| rs2248339 | Chr. 21 | T | 1.17 | 0.79 - 1.75 | 0.4327 |
| rs6809523 | Chr. 3 | A | 1.19 | 0.77 - 1.84 | 0.4377 |
| rs10842209 | Chr. 12 | A | 1.19 | 0.77 - 1.85 | 0.4388 |
| rs12865094 | Chr. 13 | C | 1.23 | 0.73 - 2.06 | 0.4424 |
| rs1150939 | Chr. 12 | C | 1.20 | 0.75 - 1.90 | 0.4448 |
| rs338586 | Chr. 19 | C | 1.17 | 0.78 - 1.75 | 0.4565 |
| rs9862758 | Chr. 3 | A | 1.23 | 0.71 - 2.11 | 0.4596 |
| rs6552131 | Chr. 4 | C | 1.23 | 0.71 - 2.11 | 0.4596 |
| rs10905165 | Chr. 10 | C | 1.17 | 0.77 - 1.77 | 0.4605 |
| rs4581094 | Chr. 8 | A | 1.20 | 0.74 - 1.96 | 0.4645 |
| rs11641649 | Chr. 16 | T | 1.16 | 0.78 - 1.73 | 0.4710 |
| rs2879097 | Chr. 17 | T | 1.19 | 0.74 - 1.94 | 0.4724 |
| rs6664617 | Chr. 1 | T | 1.18 | 0.73 - 1.91 | 0.4875 |
| rs3779078 | Chr. 7 | G | 1.19 | 0.73 - 1.93 | 0.4878 |
| rs11152695 | Chr. X | T | 1.22 | 0.69 - 2.16 | 0.4912 |
| rs4771833 | Chr. 13 | T | 1.15 | 0.77 - 1.72 | 0.4934 |
| SNP | Location | Risk allele | OR | 95% IC | P value |
| rs12091558 | Chr. 1 | C | 1.16 | 0.76 - 1.78 | 0.4990 |
| rs3779989 | Chr. 8 | C | 1.16 | 0.76 - 1.78 | 0.4990 |
| rs749691 | Chr. 2 | A | 1.16 | 0.75 - 1.80 | 0.5072 |
| rs12507821 | Chr. 4 | G | 1.16 | 0.74 - 1.82 | 0.5084 |
| rs1514579 | Chr. 5 | C | 1.16 | 0.75 - 1.78 | 0.5141 |
| rs17723088 | Chr. 11 | C | 1.17 | 0.73 - 1.86 | 0.5154 |
| rs10482973 | Chr. 21 | G | 1.20 | 0.68 - 2.11 | 0.5206 |
| rs7332804 | Chr. 13 | A | 1.20 | 0.69 - 2.09 | 0.5217 |
| rs16956634 | Chr. 15 | G | 1.17 | 0.72 - 1.90 | 0.5233 |
| rs12614009 | Chr. 2 | C | 1.18 | 0.70 - 1.99 | 0.5246 |
| rs4743646 | Chr. 9 | G | 1.15 | 0.74 - 1.79 | 0.5246 |
| rs1472272 | Chr. 7 | C | 1.19 | 0.69 - 2.07 | 0.5322 |
| rs6921533 | Chr. 6 | C | 1.15 | 0.74 - 1.79 | 0.5416 |
| rs2677744 | Chr. 15 | G | 1.14 | 0.75 - 1.72 | 0.5463 |
| rs12649113 | Chr. 4 | C | 1.17 | 0.69 - 1.99 | 0.5544 |
| rs10897976 | Chr. 11 | C | 1.16 | 0.70 - 1.92 | 0.5580 |
| rs7403350 | Chr. 15 | G | 1.25 | 0.59 - 2.65 | 0.5601 |
| rs6471309 | Chr. 8 | C | 1.13 | 0.74 - 1.73 | 0.5653 |
| rs12516015 | Chr. 5 | C | 1.12 | 0.75 - 1.68 | 0.5690 |
| rs2834297 | Chr. 21 | T | 1.12 | 0.75 - 1.68 | 0.5714 |
| rs2283023 | Chr. 7 | C | 1.17 | 0.68 - 2.02 | 0.5730 |
| rs10800928 | Chr. 1 | A | 1.16 | 0.69 - 1.93 | 0.5783 |
| rs12353283 | Chr. 9 | A | 1.16 | 0.69 - 1.95 | 0.5801 |
| rs16976432 | Chr. 15 | T | 1.25 | 0.56 - 2.77 | 0.5870 |
| rs5907245 | Chr. X | G | 1.13 | 0.72 - 1.80 | 0.5897 |
| rs4665662 | Chr. 2 | T | 1.12 | 0.74 - 1.67 | 0.5934 |
| rs6702000 | Chr. 1 | G | 1.13 | 0.71 - 1.79 | 0.5984 |
| rs4956324 | Chr. 4 | C | 1.14 | 0.70 - 1.85 | 0.5998 |
| rs3820636 | Chr. 1 | G | 1.14 | 0.69 - 1.89 | 0.6058 |
| rs2330572 | Chr. 5 | A | 1.12 | 0.73 - 1.70 | 0.6060 |
| rs8089488 | Chr. 18 | A | 1.12 | 0.72 - 1.75 | 0.6064 |
| rs4855102 | Chr. 3 | A | 1.12 | 0.73 - 1.71 | 0.6071 |
| rs2365860 | Chr. 1 | A | 1.18 | 0.62 - 2.24 | 0.6136 |
| rs10823579 | Chr. 10 | G | 1.16 | 0.65 - 2.08 | 0.6177 |
| rs12897929 | Chr. 14 | A | 1.12 | 0.71 - 1.74 | 0.6306 |
| rs13168171 | Chr. 5 | A | 1.10 | 0.74 - 1.65 | 0.6310 |
| rs7022941 | Chr. 9 | G | 1.10 | 0.74 - 1.65 | 0.6310 |
| rs6432221 | Chr. 2 | T | 1.11 | 0.73 - 1.67 | 0.6323 |
| rs1816235 | Chr. 5 | A | 1.12 | 0.71 - 1.77 | 0.6361 |
| rs7566768 | Chr. 2 | A | 1.11 | 0.71 - 1.73 | 0.6367 |
| rs4940788 | Chr. 18 | T | 1.10 | 0.74 - 1.64 | 0.6437 |
| rs4837835 | Chr. 9 | C | 1.10 | 0.73 - 1.65 | 0.6473 |
| rs2161422 | Chr. 5 | G | 1.13 | 0.67 - 1.92 | 0.6479 |
| rs17674860 | Chr. 16 | T | 1.11 | 0.70 - 1.76 | 0.6484 |
| rs4317216 | Chr. 4 | C | 1.12 | 0.69 - 1.80 | 0.6516 |
| rs7147927 | Chr. 14 | C | 1.10 | 0.73 - 1.64 | 0.6519 |
| rs9978938 | Chr. 21 | T | 1.10 | 0.73 - 1.66 | 0.6531 |
| rs9306953 | Chr. 4 | T | 1.10 | 0.73 - 1.63 | 0.6560 |
| rs2507237 | Chr. X | T | 1.11 | 0.70 - 1.75 | 0.6624 |
| rs6718080 | Chr. 2 | C | 1.10 | 0.71 - 1.70 | 0.6658 |
| rs12143535 | Chr. 1 | T | 1.12 | 0.67 - 1.85 | 0.6667 |
| rs11800122 | Chr. 1 | A | 1.12 | 0.66 - 1.89 | 0.6695 |
| rs758565 | Chr. 12 | C | 1.09 | 0.73 - 1.63 | 0.6720 |
| SNP | Location | Risk allele | OR | 95% IC | P value |
| rs11129696 | Chr.3 | A | 1.09 | 0.73 - 1.63 | 0.6735 |
| rs2072041 | Chr.1 | A | 1.09 | 0.73 - 1.64 | 0.6767 |
| rs11118329 | Chr. 1 | A | 1.09 | 0.72 - 1.65 | 0.6767 |
| rs1150798 | Chr. 6 | C | 1.13 | 0.62 - 2.06 | 0.6817 |
| rs2918630 | Chr. 2 | T | 1.09 | 0.73 - 1.62 | 0.6861 |
| rs10516050 | Chr. 5 | T | 1.11 | 0.67 - 1.85 | 0.6861 |
| rs1778025 | Chr. 1 | A | 1.09 | 0.72 - 1.64 | 0.6880 |
| rs6703590 | Chr. 1 | A | 1.10 | 0.68 - 1.78 | 0.6917 |
| rs4860662 | Chr. 4 | T | 1.12 | 0.63 - 2.01 | 0.6942 |
| rs203366 | Chr. 12 | A | 1.08 | 0.73 - 1.61 | 0.6953 |
| rs1045270 | Chr. 8 | G | 1.09 | 0.71 - 1.66 | 0.7002 |
| rs7696349 | Chr. 4 | G | 1.08 | 0.72 - 1.61 | 0.7107 |
| rs12665133 | Chr. 6 | C | 1.08 | 0.71 - 1.64 | 0.7135 |
| rs3109128 | Chr. 2 | C | 1.08 | 0.71 - 1.65 | 0.7136 |
| rs3792421 | Chr. 3 | C | 1.09 | 0.68 - 1.74 | 0.7152 |
| rs11177793 | Chr. 12 | A | 1.09 | 0.70 - 1.69 | 0.7165 |
| rs12293188 | Chr. 11 | G | 1.11 | 0.64 - 1.90 | 0.7169 |
| rs2035953 | Chr. X | G | 1.09 | 0.68 - 1.76 | 0.7186 |
| rs2042567 | Chr. 2 | A | 1.08 | 0.71 - 1.65 | 0.7199 |
| rs12452315 | Chr. 17 | C | 1.08 | 0.72 - 1.61 | 0.7208 |
| rs4886932 | Chr. 15 | G | 1.08 | 0.71 - 1.64 | 0.7224 |
| rs4711464 | Chr. 6 | T | 1.08 | 0.71 - 1.64 | 0.7224 |
| rs1968059 | Chr. 7 | C | 1.08 | 0.70 - 1.66 | 0.7293 |
| rs3779802 | Chr. 8 | C | 1.08 | 0.70 - 1.66 | 0.7324 |
| rs7666904 | Chr. 4 | T | 1.13 | 0.56 - 2.26 | 0.7353 |
| rs1980089 | Chr. 11 | G | 1.07 | 0.71 - 1.60 | 0.7397 |
| rs7298838 | Chr. 12 | G | 1.09 | 0.66 - 1.80 | 0.7415 |
| rs1514706 | Chr. 8 | C | 1.08 | 0.69 - 1.67 | 0.7417 |
| rs2144708 | Chr. 1 | A | 1.08 | 0.69 - 1.67 | 0.7448 |
| rs3811599 | Chr. 2 | T | 1.07 | 0.71 - 1.62 | 0.7491 |
| rs6919059 | Chr. 6 | G | 1.07 | 0.71 - 1.61 | 0.7520 |
| rs7604524 | Chr. 2 | T | 1.07 | 0.71 - 1.61 | 0.7597 |
| rs2546951 | Chr. 5 | T | 1.07 | 0.70 - 1.62 | 0.7597 |
| rs17563827 | Chr. 17 | A | 1.07 | 0.69 - 1.66 | 0.7622 |
| rs6546910 | Chr. 2 | C | 1.07 | 0.70 - 1.62 | 0.7650 |
| rs11612304 | Chr. 12 | T | 1.07 | 0.68 - 1.70 | 0.7665 |
| rs2878611 | Chr. 4 | G | 1.06 | 0.70 - 1.61 | 0.7755 |
| rs10512472 | Chr. 17 | T | 1.08 | 0.64 - 1.82 | 0.7762 |
| rs7229750 | Chr. 18 | G | 1.07 | 0.69 - 1.65 | 0.7789 |
| rs7073663 | Chr. 10 | A | 1.08 | 0.64 - 1.80 | 0.7802 |
| rs7099295 | Chr. 10 | C | 1.06 | 0.71 - 1.58 | 0.7818 |
| rs17095429 | Chr. 11 | C | 1.06 | 0.69 - 1.64 | 0.7856 |
| rs6468625 | Chr. 8 | T | 1.06 | 0.68 - 1.67 | 0.7884 |
| rs2275235 | Chr. 1 | C | 1.06 | 0.70 - 1.60 | 0.7967 |
| rs4659562 | Chr. 1 | G | 1.07 | 0.65 - 1.75 | 0.8018 |
| rs2955737 | Chr. 15 | G | 1.07 | 0.64 - 1.78 | 0.8074 |
| rs3848049 | Chr. 13 | G | 1.05 | 0.70 - 1.59 | 0.8112 |
| rs4921705 | Chr. 8 | C | 1.05 | 0.70 - 1.59 | 0.8112 |
| rs744166 | Chr. 17 | T | 1.05 | 0.69 - 1.60 | 0.8147 |
| rs7198956 | Chr. 16 | T | 1.07 | 0.60 - 1.89 | 0.8185 |
| rs17301249 | Chr. 6 | G | 1.06 | 0.61 - 1.84 | 0.8271 |
| rs9993426 | Chr. 4 | C | 1.05 | 0.65 - 1.70 | 0.8283 |
| rs9891018 | Chr. 17 | C | 1.05 | 0.66 - 1.67 | 0.8468 |
| SNP | Location | Risk allele | OR | 95% IC | P value |
| rs7942430 | Chr. 11 | C | 1.04 | 0.69 - 1.56 | 0.8480 |
| rs3784678 | Chr. 15 | G | 1.04 | 0.69 - 1.56 | 0.8480 |
| rs1380196 | Chr. 4 | C | 1.04 | 0.68 - 1.60 | 0.8480 |
| rs12126648 | Chr. 1 | T | 1.04 | 0.66 - 1.64 | 0.8699 |
| rs824409 | Chr. 9 | G | 1.04 | 0.62 - 1.75 | 0.8701 |
| rs2619629 | Chr. 10 | G | 1.04 | 0.62 - 1.76 | 0.8720 |
| rs4740023 | Chr.8 | T | 1.03 | 0.68 - 1.57 | 0.8749 |
| rs2886616 | Chr. 1 | A | 1.03 | 0.68 - 1.57 | 0.8773 |
| rs10739594 | Chr. 9 | T | 1.03 | 0.69 - 1.55 | 0.8773 |
| rs1203739 | Chr. 7 | A | 1.03 | 0.68 - 1.57 | 0.8828 |
| rs10515381 | Chr. 5 | C | 1.04 | 0.62 - 1.73 | 0.8854 |
| rs8090956 | Chr. 18 | T | 1.03 | 0.69 - 1.54 | 0.8877 |
| rs301177 | Chr. 3 | A | 1.03 | 0.63 - 1.69 | 0.8908 |
| rs2473545 | Chr. 6 | A | 1.03 | 0.67 - 1.58 | 0.8948 |
| rs10853004 | Chr. 17 | G | 1.03 | 0.66 - 1.60 | 0.8968 |
| rs1357119 | Chr. 2 | T | 1.03 | 0.66 - 1.60 | 0.8968 |
| rs913767 | Chr. 9 | A | 1.03 | 0.68 - 1.56 | 0.8970 |
| rs9897724 | Chr. 17 | T | 1.03 | 0.69 - 1.53 | 0.9000 |
| rs4858594 | Chr. 3 | G | 1.03 | 0.69 - 1.53 | 0.9000 |
| rs1394338 | Chr. 4 | C | 1.03 | 0.66 - 1.60 | 0.9019 |
| rs3816240 | Chr. 8 | T | 1.03 | 0.66 - 1.60 | 0.9019 |
| rs6443664 | Chr. 3 | T | 1.03 | 0.68 - 1.54 | 0.9038 |
| rs1894579 | Chr. X | C | 1.03 | 0.66 - 1.59 | 0.9058 |
| rs4653762 | Chr. 1 | C | 1.02 | 0.68 - 1.54 | 0.9078 |
| rs1414052 | Chr. 10 | T | 1.02 | 0.68 - 1.54 | 0.9119 |
| rs9514645 | Chr. 13 | G | 1.02 | 0.68 - 1.54 | 0.9119 |
| rs2271470 | Chr. 10 | C | 1.02 | 0.68 - 1.53 | 0.9186 |
| rs7919493 | Chr. 10 | G | 1.02 | 0.68 - 1.53 | 0.9186 |
| rs17298668 | Chr. 9 | T | 1.03 | 0.61 - 1.74 | 0.9212 |
| rs2147797 | Chr. 1 | T | 1.04 | 0.47 - 2.27 | 0.9304 |
| rs11123034 | Chr. 2 | A | 1.01 | 0.67 - 1.54 | 0.9492 |
| rs12447045 | Chr. 16 | G | 1.01 | 0.64 - 1.61 | 0.9497 |
| rs650436 | Chr. 11 | T | 1.01 | 0.68 - 1.51 | 0.9534 |
| rs16962723 | Chr. 16 | A | 1.01 | 0.60 - 1.72 | 0.9575 |
| rs287528 | Chr. 13 | A | 1.01 | 0.64 - 1.60 | 0.9595 |
| rs2829214 | Chr. 21 | A | 1.01 | 0.68 - 1.51 | 0.9627 |
| rs4793952 | Chr.17 | T | 1.01 | 0.67 - 1.53 | 0.9640 |
| rs10098851 | Chr. 8 | C | 1.01 | 0.68 - 1.51 | 0.9662 |
| rs1486219 | Chr. 11 | G | 1.01 | 0.58 - 1.75 | 0.9698 |
| rs1880081 | Chr. 18 | C | 1.01 | 0.49 - 2.11 | 0.9706 |
| rs10842318 | Chr. 12 | G | 1.01 | 0.55 - 1.86 | 0.9730 |
| rs1880499 | Chr. 15 | C | 1.01 | 0.67 - 1.51 | 0.9765 |
| rs10920623 | Chr. 1 | A | 1.01 | 0.62 - 1.63 | 0.9777 |
| rs6699629 | Chr. 1 | C | 1.01 | 0.65 - 1.56 | 0.9800 |
| rs10984874 | Chr. 9 | G | 1.00 | 0.65 - 1.54 | 0.9837 |
| rs12379034 | Chr. 9 | A | 1.00 | 0.59 - 1.70 | 0.9867 |
| rs2105819 | Chr. 11 | C | 1.00 | 0.67 - 1.50 | 0.9874 |
| rs10793964 | Chr. 9 | C | 1.00 | 0.65 - 1.54 | 0.9882 |
| rs10116271 | Chr. 9 | C | 1.00 | 0.67 - 1.50 | 0.9898 |
| rs517055 | Chr. 2 | A | 1.00 | 0.63 - 1.58 | 0.9913 |
| rs10895654 | Chr. 11 | T | 1.00 | 0.61 - 1.64 | 0.9979 |
| rs4585277 | Chr. 4 | T | 1.00 | 0.65 - 1.55 | 0.9982 |

SNPs are listed by p value ranking. OR: odds ratio; 95% CI: 95% confidence interval.
